# Autoimmune Hemolytic Anemia and Immune Thrombocytopenia: A Unique Presentation of Kawasaki Disease

**DOI:** 10.1155/2021/6640006

**Published:** 2021-02-27

**Authors:** Chloe Kupelian, Bindu Sathi, Deepika Singh

**Affiliations:** ^1^Department of Pediatrics, Valley Children's Healthcare, Madera, CA, USA; ^2^Department of Pediatric Hematology and Oncology, Valley Children's Healthcare, Madera, CA, USA; ^3^Department of Pediatric Rheumatology, Valley Children's Healthcare, Madera, CA, USA

## Abstract

Kawasaki disease is an acute multisystem vasculitis characterized by involvement of medium-sized vessels that mostly affects children under the age of 5 years. The presentation is typically preceded by five or more days of fever with additional clinical findings including rash, peripheral edema, mucositis, conjunctival changes, and unilateral cervical lymphadenopathy. The most feared complication of Kawasaki disease is development of coronary artery aneurysms. Common laboratory abnormalities include normocytic anemia, thrombocytosis, leukocytosis, and elevated inflammatory markers. Immune-mediated cytopenias such as autoimmune hemolytic anemia and thrombocytopenia are rarely seen at presentation in Kawasaki disease. We describe a unique case of a child presenting with autoimmune hemolytic anemia, who sequentially developed immune thrombocytopenia concerning for Evans' syndrome and eventually diagnosed with Kawasaki Disease with coronary artery dilatation. Characteristic clinical findings including extremity edema, cracked lips, and rash developed later in the course. Our patient was treated with IVIG and steroids with significant clinical improvement and complete resolution of cytopenias and coronary aneurysms on long term follow up. Timely administration of IVIG prevents and minimizes the risk of long term cardiac consequences. Hence a high index of suspicion should be maintained for this relatively common pediatric illness, even in absence of more commonly seen laboratory findings.

## 1. Introduction

Kawasaki disease (KD) is a childhood vasculitis of the medium-sized arteries with variable presentation including fever of at least 5 days, perioral mucosal changes, polymorphous rash, peripheral extremity erythema or edema, bilateral non-exudative conjunctivitis, cervical lymphadenopathy, and coronary artery changes. Laboratory values typically include a normocytic anemia, leukocytosis, and thrombocytosis [1]. Hemolytic anemia has been described after treatment with IVIG [2, 3]. Here, we report a rare case of Kawasaki disease associated with autoimmune hemolytic anemia and immune thrombocytopenia as a presenting feature prior to treatment with IVIG.

## 2. Case Presentation

A 2-year-old previously healthy, fully vaccinated male presented to the emergency room with fever of 38.5 C, fussiness, and pallor. Extensive laboratory evaluation was undertaken upon admission including hemoglobin 5.4 g/dL, hematocrit 14.7%, WBC 15.6 × 10^3/mcL, platelet count 243 × 10^3/mcL, LDH 2110 U/L, total bilirubin 3.8 mg/dL with conjugated bilirubin 0.9 mg/dL, haptoglobin <10 mg/dL, reticulocyte count 2.5%, BUN 47 mg/dL, Creatinine 0.7 mg/dL, AST 85 U/L, ALT 18 U/L, ESR 145 mm/Hr, and CRP 8.1 mg/dL. Direct Antiglobulin Test (DAT) was positive with 2+ positive cold agglutinins, negative IgG, 4+ positive polyspecific, and 4+ positive complement, consistent with pan-hemolytic, cold-agglutinin-mediated autoimmune hemolytic anemia. Peripheral smear revealed normocytic, normochromic anemia with increased polychromatic red blood cells, micro-spherocytes, and leukocytosis with infrequent atypical lymphocytes and no evidence of schistocytes, suggestive of a reactive process. Infectious labs including a respiratory viral panel, EpsteinBarr virus VCA IgG/IgM, EBV early antigen IgG, EBV nuclear antigen IgG, *Mycoplasma pneumoniae* IgG/IgM, Parvovirus B19 IgG/IgM, rapid group A *Streptococcus pyogenes* test, anti-streptolysin-O, and blood and urine cultures were all negative. Of note, this patient's case predated the discovery of novel SARS-CoV-2 virus; therefore, testing for this was not possible. On the first day of hospitalization, he required packed red blood cell (pRBC) transfusions.

On the second day of hospitalization, his hemoglobin dropped to 4.7 g/dL requiring further packed red blood cell (pRBC) transfusions. He also developed new onset thrombocytopenia with platelet count 142 × 10^3/mcL. His blood pressures were also elevated for age. Urine analysis demonstrated specific gravity 1.010, <30 protein, negative ketones, 2+ blood, negative nitrites, negative leukocyte esterase, 35 red blood cells, 19 white blood cells, 6 bacteria, and <1 casts. Repeat BUN and creatinine were 21 mg/dL and 0.66 mg/dL, respectively, and urine protein/creatinine ratio was 0.7. Additional labs including ANA, p-ANCA, c-ANCA, proteinase 3 (PR3) antibody, myeloperoxidase antibody, lupus anticoagulant, anti-cardiolipin antibody panel, and anti-beta-2-Glycoprotein I antibody panel were all negative, excluding ANCA-associated vasculitis and systemic lupus erythematosus as causes for his kidney dysfunction. Flow cytometry testing for CD59 was completed and normal, excluding paroxysmal nocturnal hemoglobinuria.

On the fourth day, the platelet count dropped to 54 × 10^3/mcL with elevated MPV 9.4 fL. The complement C3 level was 67 mg/dL and complement C4 < 2.9 mg/dL. He also developed nonbloody diarrhea and periorbital edema. With his new symptoms, there was a concern for atypical hemolytic uremic syndrome, given anemia, thrombocytopenia, acute kidney injury, hypertension, hypocomplementemia, and diarrhea. Factor H and I levels were obtained and both normal. Stool culture was negative for Shiga toxin. ADAM-TS 13 level was normal, excluding thrombotic thrombocytopenic purpura. Stool calprotectin was mildly elevated at 234 ug/g. The ferritin level obtained due to concern for hemophagocytic lymphohistiocytosis was elevated at 1365 ng/ml.

His clinical picture was concerned for possible Evans' syndrome in the setting of autoimmune hemolytic anemia and immune mediated thrombocytopenia. In light of ongoing hemolysis and cytopenias, further workup was obtained. Iliac bone marrow biopsy revealed tri-lineage hematopoiesis without malignant involvement. The bone marrow showed no evidence of hemophagocytosis, excluding hemophagocytic lymphohistiocytosis (HLH). Acute myeloid leukemia and acute lymphoblastic leukemia fluorescent in-situ hybridization (FISH) results were both normal. Given renal dysfunction, kidney biopsy was obtained which revealed acute tubular necrosis with hemoglobin-containing casts and segmental thick glomerular basement membranes. The hemoglobin casts were secondary to the ongoing hemolysis and likely tubulotoxic, leading to tubular necrosis. The significance of the thickened glomerular basement membranes is unknown. There were no features of Alport syndrome. There was no evidence of thrombotic microangiopathy, excluding atypical hemolytic uremic syndrome.

He was also evaluated for an underlying immunodeficiency syndrome. Immunoglobulin levels were all normal. CH50 (total complement) was 47 U/L, excluding total complement deficiency. Autoimmune lymphoproliferative syndrome (ALPS) testing was performed and negative.

He continued to have daily fevers. On the fifth day of hospitalization, his exam demonstrated increasing abdominal distention and tenderness to palpation. He developed pedal edema, swelling of both hands, an erythematous rash on the trunk and face, and cracked lips. An abdominal ultrasound was positive for splenomegaly, ascites, bilateral pleural effusions, and hydrops of the gallbladder. A 2D ECHO was performed, and it revealed a dilated left main coronary artery with diameter 3.70 mm (*z*-score 3.00). Given the prolonged fevers and coronary artery dilation in conjunction with rash, cracked lips, and edema, he began treatment with IVIG 2 g/kg and high dose aspirin for suspected incomplete KD according to AHA guidelines [1]. He also received a one-time pulse dosing of Solu-Medrol 30 mg/kg and maintenance dosing of 1 mg/kg twice a day thereafter.

The patient slowly began to recover with treatment. He became more alert and interactive. His hemoglobin, hematocrit, platelet count, and liver function tests all normalized. His total bilirubin, LDH, and CRP were all down-trending. One month after discharge, repeat 2D ECHO revealed normal sizes of all coronary arteries, including resolution of a previously dilated left main coronary from 3.70 mm to 2.16 mm (z-score −0.70). He remained on a prolonged steroid taper for 4 months. The patient continued to remain clinically well at greater than 1 year after initial diagnosis with normal cell counts and negative DAT. He was referred to Stanford Immunology and received genetic testing, which revealed variants of unknown significance including CARD11, FPR1, NBN, PRKDC, and TTC7A. After consulting with Immunology, none of these variants can explain his phenotype and clinical presentation. We investigated whether these variants are associated with KD; however, none of these variants have been reported in the literature in patients with Kawasaki disease.

## 3. Discussion

Kawasaki disease typically presents with leukocytosis with neutrophilic predominance, thrombocytosis, and normocytic anemia. Hemolytic anemia is a known complication of IVIG treatment; however, our patient presented with hemolysis and thrombocytopenia prior to initiation of treatment. Classic exam findings including swelling of hands/feet, cracked lips, and rash developed later on in his course. He also had other nonspecific findings including hydrops of the gallbladder with lab findings of cholestasis, which has been shown in the literature to be a nonclassical finding of KD [4].

Our patient demonstrated features of Evans syndrome, which is characterized by autoimmune hemolytic anemia and thrombocytopenia. These can occur simultaneously or develop sequentially in Evans syndrome, and several patients may also develop neutropenia [5].

We performed extensive literature review of previously reported cases of autoimmune hemolytic anemia associated with KD (Table 1) prior to treatment with IVIG [6–10]. All cases were associated with hemolytic anemia and either a normal platelet count or thrombocytosis. Thrombocytopenia in association with KD has been reported in literature [11–14] (Table 2). The pathogenesis is unclear; however, previous studies have proposed a consumptive coagulopathy [11, 15]. ITP as diagnosed by presence of immature megakaryocytes bone marrow has also been reported in the literature [16]. Previous studies have shown a greater risk of coronary artery aneurysm development in patients with KD and thrombocytopenia [17].

Furthermore, platelet count <300,000/mm^3^ is part of Kobayashi and Egami risk scoring systems developed in Japan to predict IVIG resistance [16].

In our patient, thrombocytopenia preceded aneurysmal development. Although antiplatelet antibody testing was not performed in our patient, the presence of thrombocytopenia in association with elevated mean platelet volume and increased megakaryocytes in the bone marrow is suggestive of peripheral antibody mediated platelet destruction.

While an environmental trigger in a genetically susceptible individual is suggested to contribute to development of Kawasaki Disease, a definitive infectious, environmental or another trigger has not been identified. It is suspected that both innate and adaptive arms of the immune system are implicated in Kawasaki disease. Polymorphisms in ITPKC have been reported, which in turn is thought to play a significant role in NLRP-3 inflammasome activation [18]. Activation of the adaptive immune system in the form of increased circulating regulatory T-cells has also been reported [19]. Previous studies have also indicated a role for B-cell activation in the acute phase in Kawasaki disease. Antiendothelial and antimyocardial antibodies have been described although their clinical significance is unclear at this time [20].

Features of Kawasaki disease and other autoimmune conditions such as systemic lupus erythematosus can overlap including fever, rash, lymphadenopathy, arthritis, and arthralgia [20, 21]. Patients with SLE can also develop secondary vasculitis. The role of the immune system and gene-related variations in single-nucleotide polymorphisms such as B-cell lymphoid kinase (BLK), caspase 3 (CASP3), ITPKC, FCGR2a, and CD40 has been described in both KD pathophysiology and autoimmune diseases such as SLE, ulcerative colitis, systemic sclerosis, and rheumatoid arthritis [22]. These findings taken together support the role the adaptive immune system plays in this condition. The presence of autoimmune hemolytic anemia and thrombocytopenia in our patient further supports this theory.

Several immunologic variants were present in our patient; however, none are able to explain the clinical picture seen. Mutations in CARD11 are associated with combined immunodeficiency or B cell expansion; however, our patient had both normal number of *T* and B cells. FPR1 and TTC7A are associated with periodontitis and intestinal atresias, respectively, which are both not present in our patient. PRKDC and NBN are associated with severe combined immunodeficiency and breast cancer, respectively, which are both not the case in our patient.

Evans syndrome has been reported previously after IVIG treatment for Kawasaki disease [23]. Given the development of autoimmune hemolytic anemia and immune thrombocytopenia prior to treatment with IVIG, our case is uniquely the first to describe Evans syndrome as a presenting feature of Kawasaki disease. A yet unidentified immune dysregulation cannot be conclusively ruled out in our patient.

Of note, treatment for Evans syndrome and Kawasaki disease overlaps. First-line treatment for the acute phase of KD includes high-dose IVIG and medium-to-high dose ASA. In case of refractory KD, additional therapeutic options include corticosteroids, TNF-alpha blockers such as Infliximab, and IL-1 inhibitors such as Anakinra [22]. Case reports also exist of anti-CD 20 therapy with Rituximab in refractory cases of Kawasaki disease [24].

First-line therapy for Evans' syndrome includes corticosteroids and IVIG, while second-line treatment is rituximab. If a patient fails to respond to rituximab, other immunosuppressive agents such as cyclophosphamide, azathioprine, and mycophenolate may be utilized, although the response rate varies [25]. In our patient, hemolysis and thrombocytopenia resolved after treatment with IVIG 2 g/kg (KD dosing), IV steroids, and high dose aspirin.

## 4. Conclusion

Clinicians should be aware that KD has a variable spectrum and may have autoimmune hemolysis and thrombocytopenia as presenting features. Even in the absence of more commonly seen laboratory findings, a high index of suspicion should be maintained for this relatively common pediatric illness, in light of other physical exam findings.

## Figures and Tables

**Table 1:** Literature review of articles associated with hemolytic anemia and Kawasaki disease.

Author(s) (reference)	Patient age	Features
Gupta et al. [6]	10 month	Hemoglobin 6.1 g/dL, platelet count 165,000/*μ*L, reticulocyte count 6.01%.
Jiang et al. [7]	1 year	Hemoglobin 10.4 g/dL, platelet count 314,000/*μ*L.
Thakkar et al. [8]	7 month	Hemoglobin 3.6 g/dL, platelet count 389,000/*μ*L, reticulocyte count 3.4%.
Bunin et al. [9]	7 week	Hemoglobin 11.8 g/dL, progressive thrombocytosis.
Kawasaki [10]	4 year 3 months	Hemoglobin 8.2 g/dL

**Table 2:** Literature review of articles associated with thrombocytopenia and Kawasaki disease.

Author(s) (reference)	Patient age	Presenting platelet counts
Hara T et al., [11]	10 patients mean age in years: 1.8 ± 2.6 (SD)	Mean minimal platelet count of 94000 ± 38000 (SD)/mm 3
Krowchuk et al. [12]	1 patient 33 months old	84 × 10^9^/L
Singh et al. [13]	6 patients Ages in years: 1, 1.5, 2.5, 3.8, 9, and 10	44 × 10^9^/L, 92 × 10^9^/L, 64 × 10^9^/L, 78 × 10^9^/L, 87 × 10^9^/L 40 × 10^9^/L
Asano et al. [14]	1 patient 1 year and 8 months old	75 × 10^9^/L

## Data Availability

The data used to support the findings of this study are available within the article.
